# Simulation‐Based Education Versus Traditional Teaching for Nursing Students: A Systematic Review

**DOI:** 10.1002/hsr2.72731

**Published:** 2026-07-06

**Authors:** Suleman Shah, Ameer Afzal Khan, Mohammed Al Meqbaali, Rahman Syed, Anfal Khan, Mohsin Ali, Mohammed Al Sinani

**Affiliations:** ^1^ Fatima College of Health Sciences Al Ain Abu Dhabi UAE; ^2^ Department of Medicine Saidu Medical College Swat Khyber Pakhtunkhwa Pakistan; ^3^ Department of Medicine Swat Medical College Swat Khyber Pakhtunkhwa Pakistan; ^4^ Department of Metabolism, Digestion, and Reproduction Imperial College London London UK

**Keywords:** education, learning, nursing, systematic review, teaching methods

## Abstract

**Background and Aims:**

Simulation‐based education is increasingly used in nursing programs to enhance clinical competence, yet its effectiveness compared with traditional teaching methods remains uncertain. This study aims to evaluate the effectiveness of simulation‐based education compared with traditional teaching methods in improving knowledge, clinical skills, and related outcomes among prelicensure nursing students through a systematic review.

**Methods:**

Following PRISMA guidelines, PubMed, the Cochrane Library, and ClinicalTrials.gov were searched on August 18, 2025, for studies published. Eligible studies included prelicensure nursing students who compared SBE (high/low fidelity manikins, standardised patients, virtual/augmented reality, or serious games) to traditional education. The outcomes included knowledge acquisition, clinical skills, OSCE performance, satisfaction, confidence, and retention. We considered randomised controlled, quasi‐experimental, and controlled before‐and‐after trials.

**Results:**

Thirty studies met the inclusion criteria (16 non‐randomised, 14 randomised controlled trials). SBE was generally associated with improved knowledge acquisition and retention compared with traditional methods, with notable gains in CPR, vital signs, cardiac auscultation, and paediatric nursing, particularly in randomised studies. SBE groups also demonstrated superior clinical and procedural performance, including suctioning, nasogastric tube feeding, transfusion tasks, and OSCE scores, with some effects sustained at 1 to 3 months. Learner satisfaction and self‐efficacy outcomes were variable; results generally favoured SBE in higher‐quality studies, although several reported no significant difference. GPA outcomes showed no meaningful improvement.

**Conclusion:**

The available evidence, predominantly from quasi‐experimental studies, suggests that SBE may offer advantages over traditional teaching in knowledge acquisition, clinical skills, and OSCE performance among prelicensure nursing students, though these findings should be interpreted in light of the methodological heterogeneity of included studies. Benefits on satisfaction and confidence were inconsistent. These findings support the considered integration of well‐designed simulation into nursing curricula to advance competency‐based education and patient safety.

## 1 Introduction

The development of competent and confident nursing graduates is a critical component of providing high‐quality healthcare. Traditionally, nursing education has depended heavily on lectures, classroom instruction, and supervised clinical experiences to provide students with academic knowledge and practical skills. While this model of education has produced generations of nurses, concerns have been expressed regarding the limited possibilities for safe, hands‐on training and the inconsistent nature of clinical placements in educating students for increasingly complex health‐care environments [1, 2]. In recent years, simulation‐based education (SBE) has garnered a lot of attention as an innovative educational strategy to fill these gaps.

SBE uses a range of techniques, including high‐fidelity simulators, standardised patients, virtual reality (VR), augmented reality (AR), and game‐based learning. These technologies simulate real‐world clinical situations in a safe environment, allowing nursing students to practise both technical and non‐technical skills, integrate theoretical information, and receive immediate feedback [3, 4, 5]. Simulation has been demonstrated to improve not only psychomotor competence, but also critical thinking, self‐confidence, and clinical judgement, all of which are crucial for patient safety and treatment quality [6, 7, 8]. Furthermore, certification bodies such as the National Council of State Boards of Nursing (NCSBN) have recognised SBE, which advocates for substituting certain traditional clinical hours with simulation experiences in appropriate contexts [9].

Despite the rising use of simulation in nursing curricula around the world, there are still concerns about its efficacy compared to traditional teaching methods (TMMs). Previous research and reviews yielded varying results in terms of outcomes such as acquiring knowledge, procedural skills, learner satisfaction, and confidence [10, 11, 12, 13, 14, 15]. For example, some studies found significant increases in students' knowledge scores and clinical performance following high‐fidelity simulation, while others found very moderate benefits as compared to lecture‐based teaching. This variance could be related to variations in study design, intervention reliability, and outcome measurement technique.

Given the growing investment in simulation technology and faculty training, it is critical to synthesise available research on how it compares to traditional teaching techniques. This systematic review aims to evaluate and summarise the effectiveness of SBE compared to traditional instruction among nursing students in terms of key educational outcomes such as knowledge, skills, clinical performance (OSCE), satisfaction, and self‐confidence. This review, which combines findings from randomised and quasi‐experimental studies, provides an updated and complete assessment of the current evidence base for educators, policymakers, and curriculum developers.

## 2 Methodology

### 2.1 Review Design

This systematic review was conducted in accordance with the Preferred Reporting Items for Systematic Reviews and Meta‐Analyses (PRISMA) guidelines [16]. The protocol was prospectively registered with the International Prospective Register of Systematic Reviews (PROSPERO; CRD420251150029). The research question was framed using the PICO framework:

**Population (P):** Prelicensure nursing students (undergraduate, diploma, associate degree, or Bachelor of Science in Nursing [BSN] programs).

**Intervention (I):** SBE, including high‐fidelity simulation, low/medium‐fidelity manikins, standardized patients, computer‐ and screen‐based simulation, VR, AR, and serious games.

**Comparator (C):** TTMs (didactic lectures, classroom‐based instruction, case studies without simulation, paper‐based or usual teaching).

**Outcomes (O):**

*Primary outcomes:* Knowledge acquisition (test scores) and clinical skills performance (Objective Structured Clinical Examination [OSCE], skill checklists, scenario‐based assessments).

*Secondary outcomes:* Critical thinking/clinical reasoning, self‐efficacy and confidence, satisfaction/motivation, anxiety reduction, and retention of knowledge/skills at follow‐up.

### 2.2 Search Strategy

A comprehensive search was performed on August 18, 2025 across PubMed, the Cochrane Library, and ClinicalTrials.gov for published studies. Search terms combined controlled vocabulary (MeSH) and free‐text terms for “simulation‐based education,” “traditional teaching,” and “nursing students.” Reference lists of included studies and relevant reviews were hand‐searched to identify additional eligible trials. No language restrictions were initially applied during the search; however, studies not available in English were excluded at the full‐text stage, which represents a potential source of language bias and should be acknowledged as a limitation of this review.

### 2.3 Inclusion and Exclusion Criteria

Studies were included if they involved prelicensure nursing students pursuing diploma, associate, or bachelor's degree programs and compared SBE of any fidelity, including manikin‐based, standardised patient, VR, AR, serious games, or computer‐based modalities with TTMs such as didactic lectures, classroom‐based instruction, or other non‐simulation approaches. Eligible studies were those that reported at least one measurable outcome related to knowledge acquisition, clinical skill performance, clinical reasoning, self‐efficacy/confidence, or learner satisfaction. Randomised controlled trials (RCTs), quasi‐experimental studies, and controlled before‐and‐after designs published in peer‐reviewed journals or available as theses or dissertations (grey literature) were considered.

Studies were excluded if they were conducted in practicing or postgraduate nurses or other health professions without subgroup data for nursing students, used one‐group pre‐post designs with no comparator, were completely qualitative, or were reviews, editorials, commentaries, or protocols. Studies were also omitted if they were published in non‐English languages without a translation.

### 2.4 Study Selection

Search results were exported to Zotero for citation management. Duplicate records were automatically removed. Two reviewers independently screened titles and abstracts, followed by full‐text assessment for eligibility. Discrepancies were resolved by discussion and consensus.

### 2.5 Data Extraction

A standardized extraction form was used to collect data on author, year, country, study design, sample size, type of simulation intervention, comparator, and outcome measures (knowledge, skills, OSCE performance, satisfaction, self‐confidence, GPA, follow‐up duration). Extraction was performed independently by two reviewers.

### 2.6 Data Synthesis

Given the heterogeneity of interventions and outcome measures, a narrative synthesis was performed. Studies were grouped by outcome domains (knowledge, skills, OSCE, satisfaction, self‐confidence, GPA), and mean differences or effect sizes were reported where available in primary studies. Risk of bias was formally assessed for each included study using the Cochrane Risk of Bias tool version 2 (RoB 2) for RCTs and the Risk of Bias in Non‐randomised Studies of Interventions (ROBINS‐I) tool for non‐randomised studies. Judgements were recorded as low, moderate, serious, or critical risk of bias and were integrated into the narrative synthesis when interpreting the direction and weight of evidence. All statistical measures (means, standard deviations, and where reported, confidence intervals) were extracted as reported by primary study authors. *p* values, where reported from primary studies, are presented alongside the comparison data and test used; *p *< 0.001 is reported as such, values between 0.001 and 0.01 are reported to three decimal places, and values ≥ 0.01 are reported to two decimal places, per SAMPL guidelines. As no pooled meta‐analysis was conducted, no additional statistical software was applied in this review.

## 3 Results

A total of 846 records were identified through database searches (PubMed: 646; Cochrane Library: 187; ClinicalTrials.gov: 13). After removal of 68 duplicate records, 778 records remained for screening. Following title and abstract screening, 706 records were excluded for irrelevance. The full texts of 72 reports were retrieved and assessed for eligibility. Of these, 31 were excluded due to the wrong population, 9 due to inappropriate comparison groups, and 2 due to an unsuitable study design. Ultimately, 30 studies met the inclusion criteria and were included in this systematic review (16 non‐randomised studies and 14 RCTs) [1, 2, 4, 5, 6, 7, 8, 10, 11, 12, 13, 14, 15, 17, 18, 19, 20, 21, 22, 23, 24, 25, 26, 27, 28, 29, 30, 31, 32, 33]. Study characteristics are shown in Table 1.

**Table 1:** Characteristics of included studies.

Author (Year)	Study design	Sample I/C	Intervention	Control	Outcomes
Ahmad A. Aqel (2014) [1]	RCT	45/45 = 90	HFS training	LFS CPR (AED and BLS) (Traditional training)	Pre‐ and post‐test CPR knowledge at 3 months after training
Robert D. Keegan (2015) [2]	Non randomized	32/84 = 116	Mobile simulation pre‐class active learning	Traditional reading assignment	1. Mean students' quiz scores 2. GPA assessed 3. Students questioned
Jane D. Brannan 2008 [17]	Quasi experimental	53/54 = 107	HPS	Lectures	1. Pretest and Posttest AMIQ (acute MI questionnaire) 2. Cl Tool Mean Scores by Instructional Group
El ougli (2024) [4]	Prospective Cohort	25/24 = 49	Simulation	Traditional teaching	1. Pre and post theory course test (score out of 20) for both groups, immediate post simulation, and after 30 days, test score
Jawabreh (2025) [5]	Quasi‐experimental	35/40 = 75	HFS	Traditional Lecture	1. GPA 2. Educational Practice 3. Student Satisfaction 4. Confidence.
Mehdipour–Rabori (2021) [18]	Quasi‐experimental	38/39 = 77	simulation‐based mastery learning	Traditional Lecture	1. Suction score. 2. NG Tube feeding 3. Packed cell transfusion 4. Changing fluid Box. 5. Total Score
Andreasen (2023) [19]	RCT	86/87 = 173	Desktop VR	Traditional paper‐based	1. Enough Training from ISBAR. 2. Confidence. 3. (System Usability scale (0‐100)
Ya Meng (2023) [20]	Quasi‐experimental	71/64 = 135	Instructional Videos	Traditional teaching	1. Score of theoretical assessment Objective + Subjective. 2. Score of experimental assessment
Antonia Blanie(2020) [10]	Prospective RCT	73/73 = 146	Simulation by Gaming	Traditional teaching	1. SCT score immediately and after 1 month 2. Self Assessment 3. Satisfaction
Rongmei Wang (2015) [6]	RCT	28/27 = 55	IPSE	Traditional Learning	1. Knowledge scores comparison between IPSE and Traditional Course
Wen Chang (2025) [11]	RCT (mixed methods	47/60 = 107	VRS Program	Traditional Teaching Course	1. Knowledge assessment 2. Satisfaction 3. Confidence. 4. Motivation
Caroline Stayt (2015) [7]	RCT	50/48 = 98	Simulation Based	Traditional Lectures	1. OSCE performance (of 24). 2. GPSEC (174). 3. Student Evaluation (85)
Alinier (2005) [21]	RCT	49/50 = 99	Scenario‐based simulation training	Traditional Method	1. OSCE score. 2. Confidence 3. Stress
Arikada (2023) [22]	RCT	29/24 = 53	AR tool	Traditional Half‐body training	1. Practical Skill score Mean score of performance 2. Skill assessment 3. Understanding and interest in AR tools
Vural Doğru (2020) [23]	RCT	36/36 = 72	Simulation	Control group	1. Cardiac Auscultation (knowledge) *X*(min ± max) 2. Skill comparison 3. Anxiety scores comparison
Rababa (2019) [24]	Quasi‐experimental	52/52 = 102	Branching Pathway Stimulation	TL	1. Overall CTSAS
Evrim Eyikara (2017) [25]	Quasi‐experimental	30/30/30 = 90	Sim/Sim+Lab	Lab work	1. Vital signs knowledge (BP, Respiration, and pulse). 2. Vital signs control skill
Sami M. Aloush (2008) [8]	RCT	65/66 = 131	Simulation	Lectures	Simulation pre‐ and post‐test knowledge
Uzelli Yilmaz (2020) [26]	Quasi‐Experimental	183/168	Simulation	Teaching	1. Checking nursing records 2. Verifying patient IDs 3. Informing patient 4. Safety position 5. Right documentation (IM and OP suction and Initiating IVT)
Meili Hao (2025) [12]	Quasi‐experimental	45/45	Scenario‐based simulation in combination with serialized micro‐course instruction	TTM	1. Theoretical grades 2. Practical learning grades 3. Communication 4. Self‐efficacy 5. satisfaction
Curl E.D (2016) [27]	Quasi‐experimental	59/65	Simulation + Clinical Experience	TTM	HESI Clinical Specialty Standard Exam Scores 1. Obs 2. Paeds 3. Mental health
Loai I. Tawalbeh (2017) [13]	Quasi‐Experimental	35/34	Simulation of CP Physical Exam	Traditional Learning	Confidence 1 and 30 days after 2. 6 Item questions
Miok Kim (2016) [28]	Quasi‐experimental	25/22	Simulation	Lectures	Gender role perception, Sexual Knowledge, and Sexual attitude
ALAMRANI (2018) [29]	RCT	15/15	Simulation	Traditional Teaching	Pre and post Critical thinking and self‐confidence
AhmadAyed (2022) [30]	Quasi‐experimental study	75/75	HFS	Traditional Teaching	Clinical Judgement NIRR (noticing, interpreting, responding, and reflecting).
Guichun Lin (2024) [31]	Quasi‐experimental study	40/40	High‐simulation training	TTM	1. Theory Scores 2. OSCE score 3. CTDI‐CV (overall Rating)
KirstynKameg (2010) [32]	Quasi‐experimental	17/21	HFHS	TTM	1. Self‐efficacy MVAS 1 and MVAS 2 at time point 2
Apartsakun (2025) [33]	RCT		Childbirth Learning Aid	Traditional Learning	Childbirth Learning CBLA 1. Effectiveness 2. Self‐Confidence 3. Satisfaction 4. Mean Score CBLA
Yadigar Ordu (2023) [14]	RCT	52/52	Virtual Gaming Simulation	Traditional Learning	1. Knowledge mean scores (2800 max) median (SD)
Gokcen Gokalp (2025) [15]	RCT	66/69	IMI Game‐based learning	Control group	1. IMI Checklist scores 2. Confidence in learning scales 3. Satisfaction 4. Visual Comparison scale score

### 3.1 Assessment Outcomes

Several studies evaluated assessment outcomes through quizzes, theoretical tests, or structured questionnaires. In one of the trials, high‐fidelity simulation led to significantly higher CPR knowledge both immediately and at a 3‐month interval from simulation compared with low‐fidelity training (12.67 in intervention vs. 11.22 in control) [1]. Scenario‐based simulation combined with serialized micro‐course instruction resulted in higher theoretical (80.98 in intervention, 75.02 in control) and practical learning grades (84.09 in intervention, 78.11 in control) compared with conventional methods [12]. When a cardiopulmonary physical exam was taught using simulation, students achieved greater confidence and performance scores at both baseline and 30 days (post‐test: 25.10 in intervention vs. 9.30 in control) [13]. In addition to this, Integrated patient simulation education produced higher knowledge scores (83.5 vs. 77.0) compared with traditional coursework [6]. Students who received high‐fidelity simulation training achieved superior post‐test scores on the Acute Myocardial Infarction Questionnaire (AMIQ), although differences on the AMIQ were less pronounced (15.58 ± 2.13 in intervention vs. 14.17 ± 1.86 in control) by the CI tool of instructional group. Knowledge gains were observed immediately after simulation, and still higher gains in knowledge were maintained at 30 days compared to the control [4]. No significant GPA differences were observed, and practice scores were nearly identical between groups in a 2‐week evaluation [5]. VR‐based simulation showed slight improvements in post‐test knowledge and also some modest increases in motivation, satisfaction, and confidence [11]. In another trial, simulation improved knowledge more than lectures, where post‐test scores rose from 10.5 to 21.1 in the simulation group compared with 9.8 to 19.3 in the control group [8]. Higher scores were also observed in the group with Game‐based simulation (61.27 vs. 38.8) [15]. Simulation focusing on gender role perception and sexual health knowledge improved both knowledge (0.92 vs. 0.84) and attitudes (3.79 vs. 3.54) [28]. In contrast, simulation did not significantly outperform lectures in improving critical thinking [29]. Finally, standardized testing revealed significantly higher knowledge scores in a gaming simulation group compared to controls (1798.6 vs. 1455.1) [14]. All these studies demonstrate that simulation‐based learning generally enhances assessment outcomes when compared to TTMs (TTM), with a particularly strong effect on knowledge scores and quizzes.

### 3.2 Satisfaction Outcomes

Learner satisfaction was also studied across several studies, although the results varied across studies. In one of the studies, the satisfaction scores were almost similar between simulation and traditional teaching [5]. Smaller improvements in satisfaction were also observed in VR‐based simulation (3.98–4.18) compared with controls (3.92–4.13) [11]. Whereas in another study, higher satisfaction rates were observed in a simulation group with 36 satisfied when exposed to scenario‐based micro‐courses compared control group (24 satisfied) [12]. Students participating in gaming simulations reported a higher satisfaction rate and self‐assessment scores [10]. Childbirth simulation produced marked differences, with students in the simulation group rating satisfaction much higher than control (4.84 vs. 3.20) [33]. Game‐based simulation learning also modestly improved satisfaction compared with traditional training [15]. Overall, most studies favored simulation for satisfaction, while some studies suggested equivalence between groups.

### 3.3 Confidence Outcomes

Similar to the satisfaction findings, the confidence findings also varied. Confidence scores were nearly identical between the simulation and control groups in one trial, with slightly higher scores in the control [5]. Small improvements in confidence were noted with VR simulation, though differences were minimal [11]. Desktop VR training showed a mean difference of 13.4 points in favor of simulation, though results were not statistically significant [19]. Despite higher OSCE scores, no meaningful difference in confidence was observed in the participants in one of the studies [7]. Another trial found that the pre‐test confidence scores were comparable, but no divergence in the post‐test score was observed [21]. In contrast to this, self‐efficacy scores were higher in participants receiving simulation‐based learning plus microcourse compared to traditional learning in another study [12]. Considerable increases in confidence in the simulation group were also documented in a childbirth simulation study, with a mean of 4.74 versus 3.63 in the control [33]. In addition, confidence benefits were sustained for up to 30 days following cardiopulmonary exam simulation (25 vs. 9.3) [13]. Taken together, some studies indicated meaningful confidence gains, while others found no difference, highlighting heterogeneity across interventions and measurement tools.

### 3.4 Knowledge Score Outcomes

Knowledge score gains were consistently observed among all the studies. At both immediately after simulation and 3 months post simulation, the CPR knowledge improved significantly in simulation groups [1]. In another study, Post‐test knowledge was higher in students taught via simulation compared with lectures (21.1 vs. 19.3) [8]. Simulation improved sexual knowledge and attitudes in nursing students and improved the perception among the group [28]. A standardized test revealed significantly higher scores in the gaming‐based simulation group post‐test knowledge was higher than in the control group (1798.6 vs. 1455.1) [14]. Students taught using integrated patient simulation the theoretical knowledge was superior [6]. VR simulation produced modest improvements in post‐test knowledge [11]. Simulation also enhanced both theoretical and practical knowledge of vital signs [25]. Game‐based learning improved knowledge checklist performance [15]. Theory test scores were higher following the simulation compared with controls [31]. Across the studies, simulation consistently improved knowledge across subject areas, including CPR, cardiac auscultation, vital signs, and childbirth.

### 3.5 Skill Score Outcomes

Skill‐acquiring score was one of the domains mainly affected by simulation training. Students using AR simulation achieved higher mean performance scores compared with traditional training [22]. Another study demonstrated that higher increases in Cardiac auscultation (92.0 vs. 83.0 and procedural skills (25.0 vs. 17.0) were markedly improved with simulation, accompanied by lower anxiety [23]. Simulation also produced significant improvements in vital signs skill performance across multiple cohorts [25]. Procedural mastery scores were substantially higher for suctioning, NG tube feeding, and transfusion tasks in the simulation group (141.6 vs. 109.4) [18].

These findings consistently demonstrate the superiority of simulation in enhancing practical and procedural skills.

#### 3.5.1 OSCE Score Outcomes

Simulation training yielded consistently higher OSCE results. OSCE performance was significantly higher in simulation‐trained students (18.0 vs. 13.2) compared to controls, highlighting improvement in procedural competence [7]. Similar improvements were observed both immediately and at 6‐month follow‐up (61.7 vs. 47.5) [21]. Higher OSCE scores were also reported 1 week post‐training in the simulation group, although detailed data were limited [31, 32].

#### 3.5.2 Training Scores

Several studies reported training‐specific outcomes. Total procedural training scores were significantly higher among simulation learners [18]. No meaningful difference was detected between VR and paper‐based learning for ISBAR training (absolute mean difference 0.3) [19]. Stronger performance was observed with childbirth simulation learning aids (4.66 vs. 3.62) [33].

#### 3.5.3 GPA Outcomes

Only two studies examined GPA outcomes. No significant GPA difference was observed between mobile simulation and traditional reading assignment groups [2]. GPA outcomes were also similar between simulation and lecture‐based groups in another trial [5].

Across the domains, simulation‐based learning consistently outperformed TTMs in knowledge, skills, and OSCE outcomes, with particularly stronger effects in procedural training and applied assessments. Satisfaction and confidence outcomes were more variable, with some trials demonstrating clear advantages [12, 33] while others reporting comparable outcomes [5, 7]. GPA outcomes consistently showed no benefit. Evidence from longer‐term follow‐up [1, 13, 21] suggests that simulation benefits may persist for 1 to 3 months, though few studies have extended beyond this duration.

### 3.6 Risk of Bias

**Figure 1:**
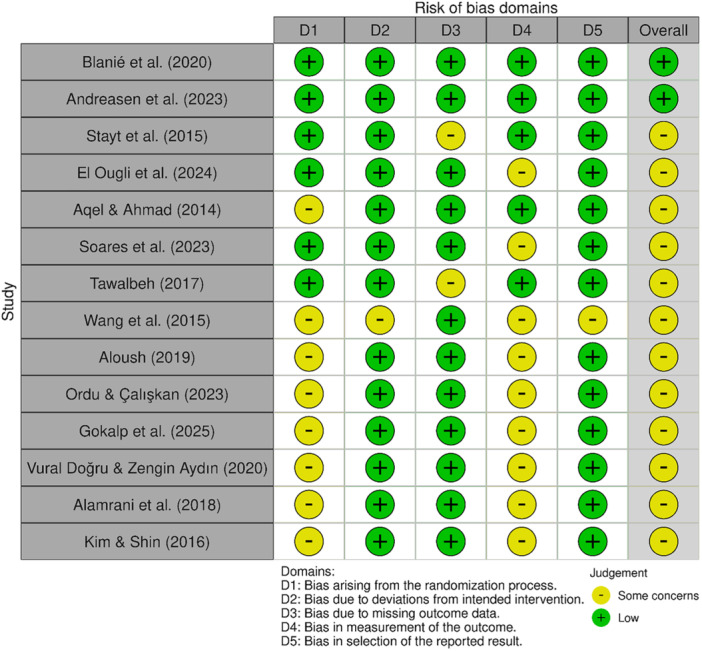
Risk of bias summary for included randomized controlled trials (RoB 2).

**Figure 2:**
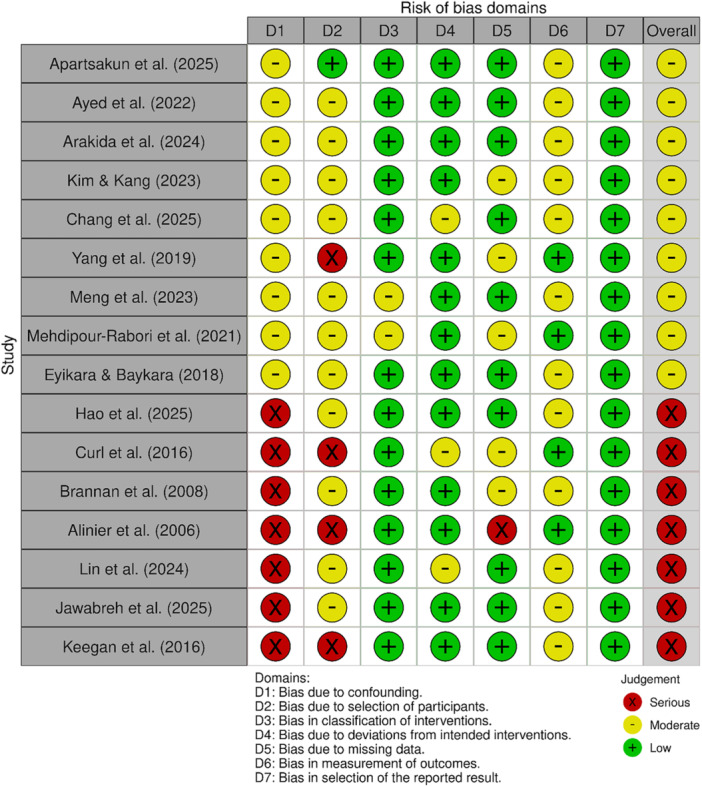
Risk of bias summary for included non‐randomized studies (ROBINS‐I).

Risk of bias was assessed using RoB 2 for the 14 RCTs and ROBINS‐I for the 16 non‐randomised studies. Among RCTs, two studies [10, 19] were rated at low risk, while the remaining 12 had some concerns, primarily due to inadequately described randomisation methods, absence of allocation concealment, and unblinded outcome assessment. Among non‐randomised studies, nine were rated at moderate risk of bias due to class‐level allocation, convenience sampling, and self‐reported outcomes, while seven were rated at serious risk, mainly due to retrospective cohort designs, institution‐level allocation, and voluntary self‐selection bias, as shown in Figures 1 and 2.

## 4 Discussion

This systematic review combines findings from 30 randomized and non‐randomized studies that compare the effectiveness of SBE with TTMs for pre‐licensure nursing students. Our results suggest that SBE is generally associated with improved knowledge acquisition, clinical skill performance, and OSCE outcomes compared with traditional teaching, although the strength of this evidence varies across outcome domains and study designs. In contrast, learner satisfaction, confidence, and GPA outcomes were more variable. These findings support the growing body of evidence that simulation is an effective instructional tool for bridging the gap between theory and practice in nursing education [9, 34, 35].

### 4.1 Knowledge Acquisition and Retention

Knowledge acquisition was the most frequently improved outcome across the included studies, though the strength of this finding varies with the methodological quality of the contributing studies. In comparison to low‐fidelity training, high‐fidelity simulation significantly enhanced CPR knowledge both immediately and 3 months later [1]. Integrated patient simulation also produced superior theoretical knowledge compared to traditional coursework [6], while scenario‐based simulation combined with micro‐courses produced higher theoretical and practical learning grades (80.98 vs. 75.02; 84.09 vs. 78.11) than conventional methods [12]. Standardised testing confirmed these advantages, with game simulation groups outperforming controls (1798.6 vs. 1455.1) [14]. These findings are consistent with previous studies that indicated significant gains in knowledge and critical thinking following SBE [36, 37]. Importantly, several studies found that SBE promotes superior recall than standard lectures [1, 12, 14].

### 4.2 Clinical Skills Performance

Our findings indicate that SBE considerably enhances clinical and procedural skill performance across multiple domains. Students educated with AR had higher mean performance scores than those taught traditionally [22], whereas cardiac auscultation and procedural performance (25.0 vs. 17.0) were considerably enhanced with simulation, which was accompanied by lower anxiety levels [23]. Suctioning, NG tube feeding, and transfusion activities demonstrated significant mastery increases (141.6 vs. 109.4) [18], and simulation improved vital signs skill performance in first‐year cohorts [25]. These findings are consistent with meta‐analyses that show that simulation offers a secure setting for repeated practice and fast feedback, resulting in increased psychomotor skill development and clinical competence [9, 36].

### 4.3 OSCE and Applied Competence

The superiority of SBE extended to OSCE performance, which is an important indicator of applied competence. Stayt et al. reported OSCE scores of 18.0 versus 13.2 in favour of simulation [7], but Alinier et al. discovered consistent improvements at 6 months (61.7 vs. 47.5) [21]. Lin et al. reported higher OSCE scores 1 week after training in the simulation group [31]. This pattern demonstrates SBE's ability to translate theoretical knowledge into practical competence and is consistent with recommendations from accrediting bodies such as the National Council of State Boards of Nursing, which supports the substitution of simulation for portions of traditional clinical hours when properly implemented [9].

### 4.4 Learner Satisfaction

Learner satisfaction outcomes were more varied. Higher satisfaction was found in scenario‐based micro‐course simulations (36 satisfied vs. 24 controls) [12], gaming simulations [10], delivery simulation (4.84 vs. 3.20) [33], and game‐based learning [15]. VR resulted in relatively minor gains (3.98 vs*.* 3.92) [11], while some investigations revealed no significant differences between groups [5]. These inconsistent results are likely due to differences in intervention fidelity, debriefing quality, or student expectations. Notably, several higher‐quality studies reported no significant benefit, and definitive conclusions about SBE superiority for satisfaction outcomes should be avoided. Previous research indicates that well‐designed situations, clear learning objectives, and organised debriefing are important predictors of learner satisfaction in simulation [38].

### 4.5 Confidence and Self‐Efficacy

Confidence and self‐efficacy outcomes also varied greatly. Significant improvements were seen in birthing simulation (4.74 vs. 3.63) [33] and cardiac assessment simulation, with confidence benefits lasting 30 days (25 vs. 9.3) [13]. Participants who received simulation with micro‐courses scored higher on self‐efficacy tests than those who received standard teaching [12]. Other studies found little or no improvement in confidence despite higher performance ratings [7, 21]. This variation may be due to differences in confidence measurement tools, exposure time, or scenario complexity. Given that several studies showed no meaningful difference, claims of SBE superiority for confidence outcomes should be made with caution. Evidence suggests that frequent simulation exposures, together with reflective debriefing, may be required to obtain long‐term self‐efficacy increases [37, 38].

### 4.6 GPA Outcomes

Unlike other domains, SBE did not show an advantage in GPA outcomes. Two studies found nearly comparable GPA scores for simulation and traditional education groups [2, 5]. GPA is likely an insensitive measure for assessing specific teaching strategies because it aggregates performance across many courses and assessment types [36].

### 4.7 Long‐Term Retention

Although most studies only provided immediate or short‐term follow‐up, several trials found that the effects of SBE on knowledge, abilities, and confidence remained for at least 1 to 3 months after training [1, 13, 21]. This suggests that SBE may not only improve immediate learning but may also support long‐term retention, which is a key factor in preparing students for clinical practice.

### 4.8 Implications for Nursing Education

These findings have substantial implications for nursing education. SBE allows students to practise high‐stakes scenarios, integrate theoretical concepts, and receive instant feedback while ensuring patient safety [9, 36]. Consistent improvements in knowledge, skills, and OSCE performance demonstrate SBE's importance in competency‐based education. However, the heterogeneity in satisfaction and confidence outcomes highlights the importance of rigorous instructional design, standardised debriefing techniques, and repeated exposures to optimise emotional learning outcomes [13, 38].

### 4.9 Limitations and Future Directions

This systematic review has several limitations that should be considered when interpreting its findings. Although risk of bias was formally assessed using RoB 2 and ROBINS‐I tools, many included studies were quasi‐experimental with small sample sizes, limited blinding, and heterogeneous outcome reporting, which lowers the overall certainty of evidence. Language bias is also acknowledged: studies without English translations were excluded despite an initially unrestricted search, and this may have introduced selective representation of the evidence. The included studies varied significantly in terms of simulation modalities (high‐fidelity manikins, standardised patients, virtual and AR, gaming), intervention duration, and outcome measures. This variability limited a proper meta‐analysis and the computation of pooled effect estimates. Furthermore, the tools employed to assess outcomes such as satisfaction, confidence, and self‐efficacy varied greatly between studies, which likely contributed to conflicting findings in these domains. Although randomised and quasi‐experimental designs were used, numerous studies had methodological constraints, such as small sample sizes, a lack of blinding, or insufficient outcome reporting, which could have introduced bias and lowered the certainty of evidence. Only a small number of studies assessed long‐term knowledge and skill retention beyond 1 to 3 months, making it difficult to conclude the long‐term effectiveness of simulation. Furthermore, despite efforts to include grey literature, publication bias cannot be eliminated, and GPA outcomes were infrequently recorded, which may not accurately reflect the influence of discrete educational interventions.

Future research should overcome these limitations by performing large, multicenter RCTs using standardised simulation techniques and validated outcome measures to strengthen the evidence foundation. Longitudinal studies are needed to assess the long‐term sustainability of simulation‐related gains in knowledge, skills, and confidence. Further research should include cost‐effectiveness assessments comparing SBE to traditional teaching techniques to help guide resource allocation and policy decisions. Finally, research into how instructional design elements such as fidelity level, structured debriefing, repetition, and integration with clinical placements affect learner outcomes, satisfaction, and self‐efficacy will provide educators and policymakers with more specific guidance on how to maximise simulation use in prelicensure nursing education.

## 5 Conclusion

Overall, this systematic review suggests that SBE may offer advantages over traditional teaching in knowledge acquisition, clinical skill performance, and OSCE outcomes for prelicensure nursing students. However, these findings must be interpreted cautiously: the majority of included studies were quasi‐experimental with small sample sizes and variable methodological quality, and high‐quality evidence from well‐powered RCTs remains limited. Satisfaction and confidence benefits were inconsistent across studies. These findings support the considered integration of simulation into nursing curricula, with priority given to well‐designed, high‐fidelity interventions accompanied by structured debriefing. Further investment in simulation infrastructure, faculty development, and methodologically rigorous research is warranted to strengthen the evidence base.

## Author Contributions

**Suleman Shah:** conceptualization, funding acquisition, software, formal analysis, project administration, supervision. **Ameer Afzal Khan:** conceptualization, writing – original draft, methodology, writing – review and editing, supervision. **Mohammed Al Meqbaali:** supervision, formal analysis, project administration, resources, writing – review and editing, writing – original draft. **Rahman Syed:** supervision, writing – review and editing, writing – original draft, visualization, formal analysis, software. **Anfal Khan:** software, writing – review and editing, writing – original draft. **Mohsin Ali:** writing – review and editing, writing – original draft, methodology. **Mohammed Al Sinani:** writing – original draft, visualization, methodology.

## AI Disclosure

No artificial intelligence tools were used in manuscript drafting, editing, data collection, literature review, or generation of any visual content in this study.

## Funding

The authors have nothing to report.

## Ethics Statement

As this study was a systematic review of previously published literature, no human participants, patient data, or biological specimens were directly involved. Therefore, ethical approval and informed consent were not required in accordance with institutional and international research ethics guidelines. The review was conducted in compliance with the principles of the PRISMA statement and was prospectively registered with PROSPERO (Registration Number: CRD420251150029).

## Conflicts of Interest

The authors declare no conflicts of interest.

## 1 Transparency Statement

Mohammed Al Sinani affirms that this manuscript is an honest, accurate, and transparent account of the study being reported; that no important aspects of the study have been omitted; and that any discrepancies from the study as planned (and, if relevant, registered) have been explained.

## Data Availability

The data that support the findings of this study are available from the corresponding author upon reasonable request.
